# HDAC Inhibitors as Epigenetic Regulators of the Immune System: Impacts on Cancer Therapy and Inflammatory Diseases

**DOI:** 10.1155/2016/8797206

**Published:** 2016-07-31

**Authors:** Elizabeth E. Hull, McKale R. Montgomery, Kathryn J. Leyva

**Affiliations:** ^1^Biomedical Sciences Program, Midwestern University, 19555 N 59th Avenue, Glendale, AZ 85308, USA; ^2^Department of Microbiology & Immunology, Arizona College of Osteopathic Medicine, Midwestern University, 19555 N 59th Avenue, Glendale, AZ 85308, USA

## Abstract

Histone deacetylase (HDAC) inhibitors are powerful epigenetic regulators that have enormous therapeutic potential and have pleiotropic effects at the cellular and systemic levels. To date, HDAC inhibitors are used clinically for a wide variety of disorders ranging from hematopoietic malignancies to psychiatric disorders, are known to have anti-inflammatory properties, and are in clinical trials for several other diseases. In addition to influencing gene expression, HDAC enzymes also function as part of large, multisubunit complexes which have many nonhistone targets, alter signaling at the cellular and systemic levels, and result in divergent and cell-type specific effects. Thus, the effects of HDAC inhibitor treatment are too intricate to completely understand with current knowledge but the ability of HDAC inhibitors to modulate the immune system presents intriguing therapeutic possibilities. This review will explore the complexity of HDAC inhibitor treatment at the cellular and systemic levels and suggest strategies for effective use of HDAC inhibitors in biomedical research, focusing on the ability of HDAC inhibitors to modulate the immune system. The possibility of combining the documented anticancer effects and newly emerging immunomodulatory effects of HDAC inhibitors represents a promising new combinatorial therapeutic approach for HDAC inhibitor treatments.

## 1. Introduction

Within a eukaryotic cell, DNA associates with histone and nonhistone proteins to form chromatin. The degree to which DNA is wound around histone proteins affects transcription: the more tightly wounded the DNA, the more condensed the DNA, and gene expression is repressed. The N-terminal regions of histone proteins are substrates for a variety of enzymes that result in posttranslational modifications of histone proteins, including phosphorylation, methylation, ubiquitination, and acetylation. Combined, these posttranslational modifications epigenetically regulate the extent of gene transcription. Of these known epigenetic factors, histone acetylation has garnered much attention in the last decade as one widely recognized factor regulating gene expression. Acetylation of histone proteins is a balance between the activities of both histone acetyltransferases (HATs) and HDACs with histone acetylation being generally associated with an increase in gene transcription while deacetylation results in decreased gene transcription.

Although it is eminently comprehensible, this simplified view leads to a vast underestimate of the effects of HDAC inhibitor treatment on chromatin structure. HDAC inhibitor treatment rapidly leads to compensating changes in histone methylation and changes in expression of histone modulators so that the effects of HDAC inhibitor treatment, even at the level of chromatin structure, are not fully delineated. In addition, as the majority of acetylation occurs on nonhistone proteins, the consequences of inhibiting HDACs using available HDAC inhibitors have profound effects on many processes independent of chromatin structure. HDAC inhibitor treatment alters gene expression at many levels including transcription factor activity, miRNA expression, and signal transduction pathways. While the most commonly reported effect of HDAC inhibitors on tumor cells is as an inducer of apoptosis, they have also been shown to interfere with cellular growth [1–3] and differentiation [2, 4] and to inhibit angiogenesis [5, 6]. In addition, HDAC inhibitors have been shown to modulate immune responses which, in turn, affect many diverse cellular functions and thus may help to explain the basis of the clinical utility of HDAC inhibitors. To harness the full potential of HDAC inhibitors, a more complete understanding of the role of acetylation on signaling at the cellular and systemic levels is required.

It is exciting that the clinical utility of HDAC inhibitors has been extended far beyond treatments for cancer, as they have now been investigated for their therapeutic potential in all top 10 leading causes of death in the US. For example, valproic acid has been used for decades for the treatment of depressive disorders with the intent to prevent suicidal behaviors (number 10 on the list) [7, 8]. More recently, HDAC inhibitors have begun to be investigated for their potential to improve outcomes following spinal cord injury, a common consequence of accidental injury (number 4 on the list) [9, 10]. In this regard, the efficacy of HDAC inhibitors appears to be contingent on their very potent anti-inflammatory actions. Indeed, the etiologies and complications which contribute to the remaining primary causes of death (heart disease, cancer, lower respiratory disease, stroke, Alzheimer's disease, diabetes, influenza and pneumonia, and kidney disease) are also inflammatory-mediated, and so discussion of HDAC inhibitors and their therapeutic, anti-inflammatory capacities is warranted.

In this review, we focus on the use of HDAC inhibitors in basic biomedical research and their impact on the immune system for several reasons. First, although four HDAC inhibitors are currently FDA-approved for the treatment of hematological malignancies, clinical trials using HDAC inhibitors against other cancers, particularly solid tumors, have failed. Our premise is that a greater understanding of the complex effects of these inhibitors on HDAC activities and cellular immunomodulatory processes is necessary in order to aid in the design of the next generation of these important drugs. Second, a more complete knowledge of HDAC enzymes and inhibitors is likely to reveal additional, still unrecognized, cellular processes which could potentially impact the pathophysiology of disease processes and must be taken into account in experimental design and in data analysis and interpretation. Finally, already proven clinically useful as anticancer treatments, HDAC inhibitors have a newly delineated role in modulating the activity of the immune system. Given the emerging importance of immunotherapy in cancer treatment, HDAC inhibitors may be useful in the development of new anticancer therapeutic strategies, including combination therapy. This review will summarize what is known about the cellular and systemic effects of HDAC inhibitors, with special emphasis as to how they relate to the immune system. But in order to achieve their full therapeutic potential, HDAC inhibitors need to be understood at all levels.

## 2. Histone Deacetylase (HDAC) Enzymes

### 2.1. Overview

Currently, 18 HDAC enzymes have been identified in mammalian cells, which are subdivided into four main classes based on their homology to yeast HDACs. Three of the four classes (Classes I, II, and IV) are zinc-dependent enzymes while Class III HDACs are NAD^+^-dependent. Class I HDACs, consisting of HDAC1, HDAC2, HDAC3, and HDAC8, are ubiquitously expressed in the nucleus in all tissues and are homologous to the yeast HDAC RDP3. HDAC1 and HDAC2 are primarily nuclear while HDAC3 and HDAC8 can shuttle in and out of the nucleus [11] and many substrates including tumor suppressors, steroid receptors, and transcription factors have been identified as substrates which are deaceylated by Class I HDACs. Class II HDACs share homology with the yeast HDAC HDA1, are associated with tissue specific functions, and deacetylate many nonhistone proteins. Class II HDACs are subdivided into Class IIA, consisting of HDAC4, HDAC5, HDAC7, and HDAC9, and Class IIB, which consists of HDAC6 and HDAC10. Class IIA HDACs show both nuclear and cytosolic localization, shuttling between these two compartments in response to different signals [11, 12]. Class IIB HDACs are localized mainly in the cytoplasm and appear to function as regulators of signal transduction and motility, as these HDACs deacetylate cortactin, Hsp90, and tubulin [1]. HDAC11 is the only member of Class IV HDACs and is homologous with Class I and Class II enzymes. Little is known about HDAC11, but expression has been noted in the kidney, brain, testes, heart, and skeletal muscle (reviewed in [13]) and has been shown to regulate oligodendrocyte development [14] and expression of interleukin-10 by antigen-presenting cells (APCs) [15]. Class III HDACs, also called sirtuins, consisting of SIRT 1–7, are homologous with the yeast sirtuin protein Sir2 (reviewed in [13]). Sirtuins are widely expressed in human tissues and regulate a variety of biological functions such as oxidative stress, DNA repair, metabolism, and aging [16, 17]. The diversity of HDACs revealed by this classification scheme suggests that HDAC inhibitors are fundamental to the regulation of numerous and diverse biological processes.

### 2.2. HDAC Activities and Multisubunit Complexes

Histone deacetylases (HDACs) are a superfamily of enzymes originally named because they remove an acetyl group from *ε*-N-acetyl lysine amino acid. Although the name HDAC implies some specificity for histones, HDACs deacetylate a wide range of nonhistone proteins (see [18–20] for a few examples) and are more appropriately termed lysine-specific protein deacetylases. These enzymes are important in the epigenetic regulation of gene expression and the control of cellular activities. It has increasingly become clear that acetylation status is a common posttranslational modification of both histone and nonhistone proteins, with 1,750 proteins (nuclear and cytosolic) identified as being regulated by posttranscriptional changes in acetylation to date [21]. In fact, phylogenetic studies have shown that histone proteins are not the primary substrates for HDACs [22], with other substrates being DNA-binding and DNA-repair proteins, transcription factors, signal transduction molecules, chaperone proteins [23–25], and proteins involved in cellular motility [19, 26] to name a few. The involvement of acetylation in a broad range of biological activities has led to intense interest in the biological roles of this posttranscriptional modification.

Understanding is further complicated by the varying interactions of HDACs with each other (e.g., associations between HDAC1, HDAC2, and HDAC3 [27, 28]) and with large, multiprotein complexes (e.g., CoREST, NuRD, Sin3, and NCoR) which, by themselves, exhibit diverse and often cell-type specific functions. For example, the NuRD complex promotes gene silencing via chromatin remodeling, while the NCoR complex is a major corepressor for nuclear receptors. Thus, inhibition of a specific HDAC may have context dependent consequences on cellular functions. In addition, it is reasonable to expect that measured IC_50_ values might differ when HDAC enzymes form complexes in cells and it is certainly possible that even the specificities of the inhibitors might be altered.

## 3. Histone Deacetylase (HDAC) Inhibitors

### 3.1. Introduction to HDAC Inhibitors

Histone deacetylase (HDAC) inhibitors are a family of natural and synthetic compounds that differ in their target specificities and activities, both in the clinical setting and in laboratory studies [25]. HDAC inhibitors are broadly classified into four main groups based on their structure: hydroxamic acids, cyclic peptides, benzamides, and short-chain fatty acids. Three of the four FDA-approved anticancer HDAC inhibitors are hydroxamic acids (SAHA, belinostat, and panobinostat) and have been reported to be nonspecific HDAC inhibitors affecting all “classical” HDACs (Classes I, II, and IV). The fourth FDA-approved HDAC inhibitor, FK228, is a cyclic peptide and has been reported to be specific for Class I HDACs. Several other HDAC inhibitors have been tested* in vitro* or are in current clinical trials and have been recently reviewed elsewhere [25, 29–32]. While generalizations regarding target specificities are valuable as a starting point, we have found that the story is much more complex. As West and Johnstone [30] point out: “… reliable determination of HDAC inhibitor target specificity* in vitro* using standard assays with recombinant HDAC proteins has been hindered due to protein misfolding, lack of enzymatic activity, and most importantly, the inappropriate assessment of isolated HDACs that exist as multiprotein complexes in physiologic conditions.”

### 3.2. Limitation in the Characterization of HDAC Inhibitors

Complete understanding of the activities of compounds in the clinical setting requires an understanding of the inhibitor effects at multiple levels, including the enzymes which may be targeted by the inhibitor, as assayed* in vitro*, in cell-based assays to determine cellular effects for broader impacts, and in animal/human studies to delineate any systemic effects revealed by preclinical and clinical trials. This portion of the review will consider the role of HDAC inhibitors at each of these levels.

#### 3.2.1. *In Vitro* Assays of HDAC Inhibitors

*In vitro* assays, usually used to definitively ascertain the mode of action of an inhibitor, have proved problematic in the case of HDAC inhibitors as the values quoted between similar assays are extremely variable. For instance, Hu et al. [33] determined, using recombinant HDAC proteins, that trichostatin A (TSA) inhibits HDAC1, HDAC3, and HDAC8 with equal potency and has IC_50_ values of 0.1–0.3 *μ*M. On the other hand, Khan et al. [11] report IC_50_ values in the nanomolar range, with TSA having lower efficacy towards HDAC8 by two orders of magnitude compared with HDAC1 and HDAC3. Similar differences were seen with MS-275 between the two studies. Although both studies expressed recombinant HDAC enzymes using the baculovirus expression system, they used different substrates. Hu et al. used purified, labeled histones while Khan et al. used a commercial fluorogenic peptide substrate. However, little difference between these two substrates was observed when HDAC enzymes purified from liver were used. Indeed, the IC_50_ values for TSA measured using isolated HDAC enzymes are similar and, more specifically, relatively similar *K*_*m*_ and *V*_max_ values for each reaction have been reported for both purified histone and peptide substrates [34, 35]. Although using HDAC enzymes purified from tissue may be deemed more physiological, these assays are unable to determine the specificity of an inhibitor. From these data, it is difficult to make conclusions as to which HDAC enzyme is targeted or what the IC_50_ values truly are for any given HDAC inhibitor as there are fundamental disagreements in the conclusions which can be drawn from each set of assays.

An alternate approach to determine specificity of HDAC inhibitors uses fluorescence resonance energy transfer (FRET) assays for measuring binding of an inhibitor to HDAC enzymes. FRET assays either directly measure the inhibitor-enzyme binding [36] or use a competition assay to measure the ability of an inhibitor to displace the compound from the active site of an experimental inhibitor-enzyme pair. Although these assays do not address how the inhibitor affects enzyme activity, they provide definitive measurements of *K*_*m*_ of inhibitor binding to each HDAC and identify possible off-target interactions. Interestingly, a similar variation in values was seen between the two assays. Marks et al. [36] report binding values of 1–10 nM for most HDAC-HDAC inhibitor pairs while Riester et al. [37] report values in the 300 nM to 1.1 *μ*M range. Only values for SAHA were measured in both assays and the values for this inhibitor range from 1.8 ± 0.3 nM in the FRET binding to HDAC6 assay and 1.0 *μ*M using the reciprocal binding constant in the FRET competition assay [36]; these and other data are delineated in Table 1.

The aggregate conclusion from these studies is that the IC_50_ of HDAC inhibitors is dependent on enzyme preparation and is also assay dependent. Without a clear understanding of the enzyme targets and potency of HDAC inhibitors, interpretation of* in vivo* work becomes more difficult and the uncertainty in the specificity, targets, and efficacy of HDAC inhibitors from* in vitro* studies must frame the interpretation of* in vivo* assays.

#### 3.2.2. Cell-Based Assays

Variation in the IC_50_ derived from* in vivo* and* in vitro* measurements is expected but this variability is exacerbated by the large number of cell-based assays used. As the effects of HDAC inhibition are wide-ranging, a variety of cell-based assays are needed to assess activity of HDACs (summarized in Table 2). Although these assays are not comparable and cannot reveal specificity, these assays have the advantage of being more biologically relevant and are better indicators of the downstream consequences of epigenetic reprogramming by HDAC inhibition.

Further investigation of HDAC inhibitor activity using cell-based assays is essential to understanding the activity of HDAC inhibitors* in vivo*. Acetylation affects the activity of a variety of nonhistone proteins and these proteins are involved in signaling and gene expression so that cellular assays of HDAC inhibitor function are essential. An apt example of this is FK228 which is FDA-approved for cutaneous T cell lymphoma. This natural product has been characterized as a relatively specific inhibitor of HDAC1 and HDAC2 (with 1–3 orders of magnitude higher IC_50_ for other HDACs tested) [38] and has been shown to induce hyperacetylation of histone H3 after 3 hours and altered p21 gene expression and cell cycle arrest after 6 hours of treatment [3]. However, FK228 has been shown to directly inhibit phosphatidylinositol 3-kinase activity in* in vitro* [42] and in cell-based assays [60]. Cellular responses to FK228 treatment include induction of apoptosis [3], increased reactive oxygen species, decreased mitochondrial membrane potential, and activation of the unfolded protein response and stress-activated protein kinase/c-Jun N-terminal kinase pathway [43]. However, as HDAC inhibitors, such as FK228, can change gene expression within hours of treatment [42] due at least in part to modification of transcription factors [25], cellular effects of HDAC treatment are difficult to predict. Although there are some commonalities in the proteins affected by HDAC inhibitors (several HDAC inhibitors will affect levels of p21 to trigger apoptosis), the cellular targets of most HDAC inhibitors are varied and sometimes unexpected.

#### 3.2.3. Multiple Targets for Most HDAC Inhibitors

Unfortunately, many commercially available HDAC inhibitors have not been sufficiently well characterized to support use in the manner for which they are marketed. For instance, depudecin is a natural product which was first identified as a compound that reversed transformation by the* ras* oncogene [61] but was later identified as an HDAC inhibitor [4]. Although this HDAC inhibitor is marketed as an HDAC1 inhibitor, depudecin has only been tested for activity against HDAC1 using an* in vitro* recombinant assay [4] so that its true target specificity profile has not been determined. Similarly, MC1293 is marketed as an HDAC1 inhibitor but a substrate independent FRET assay of inhibitor to protein binding suggests that this inhibitor binds to HDAC1 with low affinity while binding to HDAC6 with two orders of magnitude greater affinity [36].

Many* in vitro* assays of Class I HDAC inhibitors focus on histone targets and as Class I HDACs are localized to the nucleus, this seems to be a reasonable strategy. However, recent advances in mass spectrometry techniques have revealed that this ancient form of protein regulation is intimately linked to metabolism and that acetylation may affect gene expression at multiple levels (reviewed in [62]). The consequences of acetylation are far-reaching. For instance, acetylation changes the activity of transcription factors (e.g., p53, HIF1a, NF-*κ*B, EKLF, E2F1, STAT1, GATA1/2/3, SrY, and MyoD) (reviewed in [25]). However, many consequences are downstream of transcription factors and are therefore more difficult to characterize. For instance, treatment with HDAC inhibitors changes histone methylation [63, 64], adding to the effect of acetylation on the binding of chromatin regulatory complexes (reviewed in [62]). Thus, much more characterization of the targets and consequences of acetylation must be completed.

### 3.3. Effects of HDAC Inhibitor Treatment

In highlighting the role of HDAC inhibitors as master epigenetic regulators, it is remarkable how cells treated with HDAC inhibitors are capable of self-regulation, ensuring that global changes are not lethal. Cells, and even entire organisms, can somehow tolerate extensive hyperacetylation of core histones and other proteins that occur following treatment. Recent work has demonstrated that cells respond to HDAC inhibitor treatment by rapidly increasing H3K27me3 at the transcription start sites of genes capable of slowing growth, as well as minimizing protein hyperacetylation until gene expression patterns can be restored [65]. Once cells have adapted to survive this initial epigenetic disruption, HDAC inhibitors can further influence gene expression by other forms of epigenetic control, including indirectly impacting DNA and histone methylation, manipulating polycomb group proteins and proteins within the SWI/SNF complex, and regulating miRNA expression. Specific examples of how HDAC inhibitors can epigenetically regulate gene expression to influence desirable cellular outcomes are described below and summarized in Table 3.

#### 3.3.1. Gene Expression

Regulation of histone acetylation by the activity of histone acetylases and histone deacetylases is a fundamental step in the epigenetic control of gene expression. Surprisingly, in spite of this seemingly generic global control of gene expression, only about 5–10% of gene transcription has been shown to be influenced by HDAC inhibitor treatment, with approximately half of the affected genes being upregulated and half being downregulated. However, HDAC inhibitors also mediate changes in gene expression via a direct effect on the activity of transcription factors and on multiple modes of epigenetic control; it is this level of reversible epigenetic regulation that makes HDAC inhibitors attractive and potentially effective manipulators of cellular and immunological outcomes.

Understanding the pleiotropic effects of HDAC inhibitors at the cellular level is complicated by the fact that HDACs can form complexes. Intriguingly, though SAHA and BML-210 are described as pan-HDAC inhibitors, the aminobenzamide BML-210 demonstrates a higher selectivity for the HDAC3-NCoR complex than its hydroxymate counterpart, which demonstrated higher levels of selectivity for HDAC1 and HDAC2 [48]. This work also demonstrated that aminobenzamides have a much smaller effect on the Sin3 protein complex than HDAC1/HDAC2 containing CoREST and NuRD complexes. Recently, these relationships between HDAC inhibitors and cellular complexes have been elegantly mapped by Bantscheff et al. [48]. Clearly, the cellular activity of each HDAC inhibitor is more complex and cannot be predicted from knowledge of the HDAC to which it binds.

Preventing or reducing transcription factor deacetylation represents another mechanism by which HDAC inhibitors have been shown to influence gene expression. For example, HDAC inhibitor-promoted acetylation stimulates the DNA-binding activity of p53, resulting in enhanced proapoptotic gene transcription [66]. Conversely, HDAC inhibition decreases angiogenic gene expression by decreasing HIF-1*α* stability and transcriptional activity [5]. Notably, nonhistone protein acetylation can also influence protein stability, localization, and enzymatic activity, as well as interaction with other proteins.

#### 3.3.2. HDAC Inhibitors as Epigenetic Regulators

*(1) Chromatin Remodeling Enzymes*. HDAC inhibitor treatment results in differential expression of epigenetic regulators such as chromatin remodeling enzymes. For example, enhancer of zeste homolog 2 (EZH2), a component of the polycomb-repressive complex involved in gene silencing and H3K27me3, is increased in aggressive tumors, but it is inhibited by HDAC inhibitor treatment [91]. Decreased EZH2 expression results in the demethylation, and subsequent reexpression, of E-cadherin, which is associated with a much less aggressive cancer phenotype [91]. Similarly, HDAC inhibitors have been proven to be effective in restoring the expression of the mutually exclusive, catalytically active subunits of the SWI/SNF complex, BRM and BRG1, which are silenced in nearly 20% of all cancers [92–94]. However, the therapeutic utility of restoring BRM and BRG1 expression via HDAC inhibition is still in question as* in vitro* work has demonstrated that while reexpression of these proteins does slow cancer cell growth, it also promotes metastatic traits [63].

*(2) Methylation*. HDAC inhibitor treatment leads to a rapid change in histone and DNA methylation. In pulmonary fibroblasts, hypermethylation of the Thy-1 promoter region results in decreased Thy-1 expression and enhanced fibrosis. Treatment of Thy-1 (−) rat fibroblasts with the HDAC inhibitor TSA significantly decreased DNA methylation while coordinately increasing H3K4me3 in the promoter region of Thy-1, resulting in restored Thy-1 expression [95]. However, in skeletal muscle fibroblast cells obtained from patients with Huntington's disease, HDAC inhibitor treatment resulted in differential effects on DNA methylation status, with ~62% of all promoter regions assessed becoming demethylated, while 48% had increased methylation [96]. Following up on this work* in vivo* using mice with Huntington's disease phenotype, the researchers also observed that these changes in methylation status were associated with improved motor function and cognition in mice treated with an HDAC inhibitor compared to mice treated with a vehicle control [96].

*(3) miRNA*. The regulation, and dysregulation, of miRNA in human health and disease also demonstrates the utility of HDAC inhibitors in the epigenetic control of cellular gene expression as miRNA expression is also altered by HDAC inhibitor treatment. miRNAs are small noncoding RNA molecules that posttranscriptionally repress target gene expression. HDAC inhibition can rapidly alter miRNA levels and in turn indirectly mediate miRNA target gene expression. For example, treatment of breast cancer cells with a combination of sodium butyrate and panobinostat increased the expression of miR-31 which was accompanied by a subsequent repression of its target protein BIM1 that resulted in the induction of cellular senescence [52]. A study investigating the effects of HDAC inhibitors in hematopoietic malignancies revealed that HDAC inhibition relieves Myc-mediated transcriptional repression of the miR-15 and let-7 miRNA families, allowing for cells to downregulate the expression of the antiapoptotic genes Bcl-2 and Bcl-xL and, thus, induce apoptosis [44].

HDAC inhibitors also have the potential to decrease miRNA abundance on a much broader scale as HDAC1 has been shown to enhance miRNA processing by acetylating the miRNA processing protein DGCR8, which increases the production of mature miRNA species [97]. However, not all HDACs have this same effect, and some miRNAs appear to be more responsive than others [97]. Notably, several HDACs are also targeted by miRNA, which means that miRNAs also have the capacity to act as HDAC inhibitors. For example, miR-449a regulates prostate cancer cell growth and viability in part by targeting and repressing HDAC1 [98]. In hepatocellular carcinoma, overexpression of miR-221 has been proposed to result in the chronic inactivation of its tumor-suppressor target, HDAC6, thereby promoting liver tumorigenesis [99]. These are just a few examples of HDAC-mediated epigenetic regulation that highlight the potential power of using HDAC inhibitors for therapeutic purposes, but harnessing this potential will be hampered until we can truly understand how they work.

### 3.4. Summary of HDAC Inhibitors and Gene Expression

The first clinical success regarding use of HDAC inhibitors was seen in the treatment of certain forms of cancer, most notably cutaneous and peripheral T cell lymphomas. Development of these Class I-specific HDAC inhibitors focused on their ability to change gene expression, which subsequently led to inhibition of cell growth and apoptosis. This early success was not recapitulated in clinical trials using HDAC inhibitors for the treatment of solid tumors [100–102] which helped in leading to the refocusing of attention to the role of HDAC inhibitors as epigenetic modifiers. However, the dichotomy between liquid and solid tumors highlights the question of why hematopoietic tumor cells appear to be more susceptible to HDAC inhibition than normal cells but there is no shown efficacy of HDAC inhibitors against solid tumors. The answer as to why is unknown but may be related to cellular differences in the epigenomic changes in acetylation patterns that may alter the balance of pro- versus antiapoptotic protein expression [30], changes in transcription factor activity [90, 103, 104], or change in the expression of immunomodulatory proteins (see Table 4).

## 4. Impact of HDAC Inhibition on the Immune System

As delineated above, HDAC inhibitor treatment leads to diverse effects via multiple mechanisms to influence pathways which affect immune function. In aggregate, these signals may result in modulation of immune function and impact the role of the immune system in a variety of diseases, including cancer. Knowing that many of the underlying immune mechanisms that are activated to fight tumor cells are also important in regulating immune responses, it comes as no surprise that HDAC inhibitors have been studied for their utility in treating chronic inflammatory disorders [119].

### 4.1. Direct Impacts of HDAC Inhibitors on the Immune System

Early studies examining the influence of HDAC inhibitors on the immune response led to mixed results. In a mouse model of systemic lupus erythematosus (SLE), treatment with TSA resulted in decreased mRNA expression of several inflammatory cytokines, including IL-6, IL-12, and IFN-*γ*. What is interesting is that decreased mRNA expression of IL-10, which is a major anti-inflammatory cytokine, was also noted [106]. Earlier work using peripheral T cells isolated from SLE patients also showed a similar effect of decreased mRNA expression of IL-10 but an increase in IFN-*γ* was also seen [107]. Two other early studies using animal models for rheumatoid arthritis have demonstrated that treatment with TSA, phenylbutyrate [46], or FK228 [45] resulted in a decrease in the proinflammatory cytokine TNF-*α*. Since this initial work, numerous studies from both human and animal models of inflammatory or autoimmune diseases have documented the anti-inflammatory properties of several HDAC inhibitors [105, 115, 119–124]. While the anti-inflammatory nature of many HDAC inhibitors is well documented, this effect appears to be dose-dependent. Leoni et al. [49] documented that the dose of SAHA needed to exert its antitumor effect was much higher (1–5 *μ*M) than lower doses (50–200 nM) that resulted in a reduction in the production of the proinflammatory cytokines TNF-*α*, IL-1*β*, IFN-*γ*, and IL-12. As reviewed in [120], several studies have corroborated that lower doses of HDAC inhibitors are anti-inflammatory, resulting in a decrease in proinflammatory cytokine production. This is not the case for all HDAC inhibitors; however, Cantley et al. [105] showed that the anti-inflammatory effects of MS-275 on human osteoclasts were only significant at the higher doses tested. These, and other studies, highlight the need for careful study design, taking into account cell type, dosage, and conservative data interpretation on the effectiveness of HDAC inhibitors as anti-inflammatory agents.

### 4.2. Anti-Inflammatory Uses of HDAC Inhibitors in Treatment of Chronic Inflammatory Diseases

In recent years, the use of HDAC inhibitors for the treatment of chronic inflammatory diseases has gained considerable attention. Two HDAC inhibitors (TSA and SAHA) have shown promise in future treatment for type 1 diabetes, a metabolic disease which has a substantial inflammatory component. In an* in vitro* study using pancreatic beta cells, both TSA and SAHA reduced cytokine-mediated cellular destruction in an NF-*κ*B dependent manner, indicative of a reduction in the inflammatory pathology [125]. In other immune-related pathophysiological mechanisms, TSA has been shown to reduce production of proinflammatory cytokines and ameliorate pathological destruction of myelin in a murine model of multiple sclerosis [126–128]. However, for another HDAC inhibitor (sodium butyrate) examined in models for multiple sclerosis, increases in MMP-9 were observed, which is associated with a proinflammatory process [129]. Thus, a careful assessment of the pro- and anti-inflammatory effects of HDAC inhibitors needs to be characterized.

### 4.3. HDAC Inhibitor Effects on Monocytes and Macrophages

As a key regulator of immune responses, monocyte and macrophage responses to HDAC inhibitor treatment are of particular importance. Human monocytes stimulated with proinflammatory lipopolysaccharide (LPS) or TNF-*α* and subsequently treated with the novel Class I HDAC1 inhibitor NW-21 decreased synthesis of the proinflammatory cytokines MIP-1*α* and MCP-1, suggesting that HDAC inhibitor treatment may help reduce synovial inflammation and be potentially useful in the management of rheumatoid arthritis [105]. It is interesting to note that levels of other proinflammatory cytokines, notably TNF-*α* and IL-1*β*, were not affected [105] when treated with NW-21. Finally, in both* in vitro* and* in vivo* models for inflammatory bowel disease (IBD), both valproic acid and SAHA drastically reduced TNF-*α* and IFN-*γ* levels, suggesting that HDAC inhibitors may prove fruitful as a novel therapy for the treatment of IBD (as reviewed in [122]).

Taken together, these studies reveal that a greater understanding of the specific cellular and molecular effects of HDAC inhibitors in mediating inflammation is sorely needed and will enhance their utility in the treatment of inflammatory diseases. These required future studies include the delineation of the pathways involved in immune modulation, either pro- or anti-inflammatory, of HDAC inhibitor treatment in a variety of experimental systems.

## 5. The Power and Pleiotropy of HDAC Inhibitors as Epigenetic Regulators

The reversible nature of epigenetic aberrations contributing to human diseases makes them desirable therapeutic targets. As such, HDAC inhibitors, which can act as epigenetic rewriters, are particularly attractive therapeutic tools. However, epigenetic processes are susceptible to both inherited and environmental conditions and are often cell-type specific, making their interpretation difficult for both researchers and clinicians alike. Furthermore, though there is certainly evidence to suggest that epigenetic aberrations may underlie many disease pathologies, there is also evidence to suggest that epigenetic changes may arise from subclinical influences (e.g., diet and age) or may be secondary to other diseases processes (e.g., inflammation), leading to the question of causality. Some illustrations of the challenges associated with the use of HDAC inhibitors as epigenetic regulators are highlighted below.

For example, HDAC inhibitor treatment of two colon cancer cell types, HCT116 and HT29, revealed differential changes in gene expression profiles as assessed by microarray analysis [130]. Similar differential effects have also been observed in miRNA expression levels following HDAC inhibitor treatment as increases in miRNA expression have been noted in colorectal cancer and lymphoma cell lines, but no changes in miRNA levels were observed following TSA treatment of the A549 lung cancer cell line [44, 131, 132]. The differences in miRNA expression in the colorectal cell lines were methylation-dependent, while the increases in miRNA expression in lymphoma lines were dependent upon Myc acetylation and transcriptional activation [44, 131], so it is likely that pretreatment methylation and acetylation status are critical to HDAC inhibitor function.

Interestingly, the Myc-mediated control of miRNA in lymphoma cells was dependent upon cellular transformation. In untransformed cells, Myc transcriptionally activates the miR-15 and let-7a families leading to the repression of miRNA targets Bcl-2 and Bcl-xL, triggering apoptosis, while this pathway is inactive in transformed cells [44]. However, in hematopoietic malignancies, HDACs contribute to the repression of these miRNAs by preventing Myc-dependent transcriptional activation. The use of HDAC inhibitors, specifically HDAC3 inhibitors, relieves the transcriptional repression of the miR-15 and let-7 families, effectively triggering apoptosis and killing the cancer cells [44].

However, while apoptosis and growth inhibition are almost ubiquitously observed in all cancer cells when treated with HDAC inhibitors, other studies have shown that many cell types will also develop metastatic properties. For example, Lin et al. reported that HDAC inhibitors enhanced metastatic properties in 13 of 30 human cancer cell lines tested [133]. These cell types could be precategorized into two distinct phenotypes, but factors contributing to this undesirable HDAC inhibitor responsiveness are unclear. Intriguingly, one cell type that was unaffected by HDAC inhibitor treatment was the A549 lung cancer cell line in which miRNA expression was reported to be unchanged in response to HDAC inhibition, making it interesting to speculate as to the role of miRNA in HDAC inhibitor responsiveness.

In addition to cancer therapeutics, the successful use of HDAC inhibitors to target epigenetic dysregulation in psychiatric disorders and findings that HDAC inhibitors can function as potent anti-inflammatory agents have recently sparked much intrigue as to the possibility of using HDAC inhibitors to treat or even prevent neurodegenerative disorders as well. Additionally, evidence suggests that cognitive aging deficits are a result of disrupted epigenetic regulation and a decreased capacity to deal with inflammatory events [134, 135]. So it was exciting when researchers demonstrated that HDAC inhibitor administration can improve age-associated memory impairment in rodents. However, follow-up work revealed that neural plasticity declines with age and that treatment is ineffective in aged rodents. Furthermore, in young rodents, previous memory training had a significant influence on the effects of HDAC inhibitors on neural gene and protein expression [135] leading the authors to conclude that the effectiveness of HDAC inhibitors on neuroepigenetic control “may vary widely in association with an individual's unique history and ongoing experience [135].”

This type of scenario brings up another significant challenge in the field of epigenetic therapeutics, which is the identification of definitive epigenetic biomarkers and therapeutic targets. For example, the use of epigenome studies has shown that DNA methylation status is associated with CVD risk, but so are things like age, obesity, air pollution, and smoking, which in turn can also influence DNA methylation status [136]. Without demonstration of causality it is not possible to differentiate disease from epiphenomenon. Thus, even though candidate biomarkers have been used successfully in research settings, their utility in a clinical setting remains unclear.

## 6. New HDAC Inhibitor-Based Therapeutic Approaches

### 6.1. Combination Therapies with Cancer Chemotherapeutics

Hematologic malignancies have been successfully treated with HDAC inhibitors, albeit with a relatively low therapeutic efficacy. Yet, HDAC inhibitors have generally been less effective against solid tumors, with cancer progression being observed in solid tumors following HDAC inhibition. In a study to investigate why this might occur, Lin et al. observed that HDAC inhibition resulted in the development of a metastatic phenotype in 43% of the 30 human cancer cell lines tested [133]. As 90% of cancer-related mortality is the result of metastasis and invasion of tissues that are distant from the primary site, these findings warrant continued investigation [137]. Lin et al. were able to demonstrate that HDAC inhibitor-enhanced cell migration and metastasis could be suppressed by cotreatment with the protein kinase C inhibitors tamoxifen and curcumin [133]. Thus, successful treatment of solid tumors with HDAC inhibitors may require the use of combination therapies and several are currently in clinical trials (reviewed in [138]). Indeed, promising clinical outcomes have been observed in the treatment of metastatic non-small cell lung cancer when SAHA treatment was combined with carboplatin and paclitaxel, as well as in breast cancer patients when MS-275 was combined with an aromatase inhibitor [139, 140].

### 6.2. Immune and Anticancer Combination Therapies: The Melanoma Example

HDAC inhibitors have dual actions in the treatment of melanoma. Several groups have shown impaired proliferation characteristic of HDAC inhibitor treatment in melanoma cell lines [112, 114, 141] and have gone on to demonstrate that disruption of HDAC activity resulted in altered expression of MHC and costimulatory molecules [118, 141]. The subsequent increase in immunogenicity results in increased activation of T cells and prolonged survival in animal models [112, 114, 117, 118]. Three of these groups have presented strong evidence that inhibition of HDAC6 plays a critical role in the events leading to increased activation of naïve T cells [112, 117, 141]. It should be noted that improved immunogenicity and immune surveillance are not limited to melanoma [142]. Manning et al. have shown that TSA leads to the reexpression of MHC Class I after upregulation of proteins involved in antigen processing and presentation in HPV 16 associated tumors [113].

However, as with other examples of the clinical uses of HDAC inhibitors, the effects of HDAC inhibition may result in opposing effects. Although abrogation of HDAC6 in macrophages and dendritic cells results in diminished production of IL-10 and induction of inflammatory APCs that activate naïve T cells [117], inhibition of HDAC11 leads to increased IL-10 production [116]. Similarly, inhibition of SIRT 1 strengthens the suppressive activity of Tregs and may be useful in enhancing Treg-based therapeutic approaches to autoimmune diseases or graft rejections [90]. In a melanoma specific example, HDAC inhibition upregulates expression of PD-1 ligand, which in and of itself would promote tolerance and decrease immune surveillance. However, this also makes cells more susceptible to immunotherapy with the anti-PD-1 receptor antibodies pembrolizumab and nivolumab [110].

## 7. Conclusions

This review has highlighted just some of the tremendous sources of variability one must consider during experimental design and selection of an HDAC inhibitor for experimental use. Typical of any inhibitor-based study, it is important to consider not just the desired class of HDAC targets, but also the concentration of the inhibitor and the type of inhibitor being investigated. However, because of the variable functions of HDAC inhibitors on multiple pathways, responses to treatment with HDAC inhibitors, including alterations in gene expression, are likely to vary not only from individual to individual, but also from cell type to cell type within an individual. Therefore, each individual patient might respond differently to treatment for a number of reasons, and the continued investigation into molecular mechanisms underlying HDAC inhibitor function is of utmost importance as the therapeutic potential of HDAC inhibitors appears to be almost unlimited.

In summary, HDAC inhibitors have been used with varying degrees of success* in vitro*,* in vivo*, and in clinical settings to treat cancer, neurodegenerative diseases, and immune disorders, and even as combinatorial approaches for antiviral therapy. However, due to the pleiotropic nature of HDAC inhibitors, modes of action, and their interactions with large numbers of proteins, it is almost certain that a single mechanism of action for a given HDAC inhibitor will never be described. Furthermore, the gene signature of the cell, as well as the cellular environment, can greatly affect the response to HDAC inhibitor treatment. Thus, as we continue to investigate the therapeutic potentials of HDAC inhibitors, it will likely be helpful to first perform comprehensive chemoproteomic profiling as the action of HDAC inhibitors should be considered in the context of the multiprotein complexes they target and not as purified catalytic subunits. Not only does this methodology allow for the identification of off-target effects, but also it supports our understanding of the function of individual HDAC inhibitors in a greater biological context in order to better predict clinical efficacy.

## Figures and Tables

**Table 1:** Specificities and IC_50_s for Class I HDAC inhibitors (HDACi).

HDACi	HDAC1	HDAC2	HDAC3	HDAC8	Purified HDACs	Additional HDACs
TSA (trichostatin A)	2 ± 0 nM [11] 19 ± 0.9 nM [38] 100–300 nM [33]	3 ± 0 nM [11] 19 ± 0.9 nM [38]	4 ± 1 nM [11] 100–300 nM [33]	~100–300 nM [33] 456 ± 59 nM [11]	2.4 ± 0.5 nM [2] 3.4 nM [39]	Active against Class II HDACs (HDAC4 6 ± 2 nM, HDAC6 3 ± 1 nM, HDAC7 5 ± 2 nM, HDAC9 6 ± 5 nM) [11] HDAC4 80 ± 18 nM, HDAC6 14 ± 4.3 nM [38]

SAHA (vorinostat)	21 ± 13 nM [40] 68 ± 14 nM [11]	164 ± 45 nM [11]	37 ± 11 nM [40] 48 ± 17 nM [11]	1200 ± 38 nM [40] 1524 ± 463 nM [11]	70 ± 40 nM [40]	Activity against HDAC6 HDAC6 0.025 ± 0.006 nM [40]

MS-275 (entinostat)	180 ± 70 nM [40] 181 ± 62 nM [11] ~300 nM [33]	1155 ± 134 nM [11]	740 ± 250 nM [40] 2311 ± 803 nM [11] ~8000 nM [33]	>10,000 nM [11] 44,900 ± 18,100 nM [40] >100,000 nM [33]	11,000 ± 1,800 nM [40]	Minimal activity against Class II HDACs HDAC6 >100,000 nM [40] HDAC9 505 ± 37 nM [11]

MGCD0103 (mocetinostat)	34 ± 17 nM [11] 82 ± 1.4 nM [40] 150 ± 20 nM [1]	34 ± 8 nM [11] 290 ± 80 nM [1]	620 ± 160 nM [40] 998 ± 431 nM [11] 1660 ± 690 nM [1]	1850 ± 1060 nM [40] >10,000 nM [1, 11]	≥25,000 nM [40]	Minimal activity against Class II HDACs HDAC11 590 ± 230 nM [1]

FK228 (romidepsin)	1.6 ± 0.9 nM [38] 4 nM [41]	3.9 ± 2.7 nM [38]		40 nM [41]		No documented activity against Class II HDACs

**Table 2:** Assays to assess HDAC inhibitor effects.

Cell-based assays	References
Apoptosis	[1, 33, 42–44]
Cell cycle arrest	[1, 3, 12, 45, 46]
Cytotoxicity	[40, 41, 47]
Differentiation	[2, 4]
Gene expression	[1, 3, 33, 40]
Growth arrest	[2, 3, 11]
Gene expression changes	[1, 3, 33, 40]
Histone hyperacetylation	[1, 3, 12, 39, 40, 47, 48]
Inflammation	[48–50]
Motility	[1, 19, 26]
ROS induction	[43, 51]
Senescence	[52–54]
Viability	[1, 12, 40]

*In vivo* assays	References

Antiangiogenesis	[5, 6]
Drug seeking behavior	[55]
Oncogenesis	[1, 40, 56, 57]
Senescence	[58]
Thermogenesis	[59]

**Table 3:** Effects of acetylation of nonhistone proteins by HDAC inhibitors.

Nonhistone proteins	Classification of protein	Function of acetylation	References
		Increased DNA binding affinity	[66]
p53	Tumor suppressor	Increased transcriptional activation	[66–68]
		Increased protein stability	[68, 69]

RUNX3	Tumor suppressor	Increased transcriptional activation	[70]
		Increased protein stability	[70]

		Increased DNA binding affinity	[70–72]
STAT3	Signaling mediator	Increased transcriptional activation	[71, 72]
		Promotes protein-protein interaction	[71, 72]

*β*-catenin	Signaling mediator	Promotes nuclear localization	[73]

		Increased transcriptional activation (basal)	[74]
Estrogen receptor	Steroid hormone receptor	Decreased transcriptional activation (ligand dependent)	[74]
		Increased protein stability	[75]

Myc	Transcription factor	Increased protein stability	[76, 77]

EKLF	Transcription factor	Promotes protein-protein interaction	[78]
		Increased transcriptional activation	[79]

		Increased DNA binding affinity	[80, 81]
E2F1	Transcription factor	Increased transcriptional activation	[80, 81]
		Increased protein stability	[80]

GATA family	Transcription factor	Increased DNA binding affinity	[82, 83]
		Increased transcriptional activation	[82–84]

HIF-1*α*	Transcription factor	Decreased transcriptional activation	[85]
		Decreased protein stability	[85]

MyoD	Transcription factor	Increased DNA binding affinity	[86]
		Increased transcriptional activation	[86–88]

NF-*κ*B	Transcription factor	Disrupts protein-protein interaction	[89]

Foxp3	Transcription factor	Increased stability	[90]

**Table 4:** Changes in expression of immune-related genes in response to HDAC inhibition.

MCP1 (monocyte chemotactic protein 1)	[105]
MIP-1a (macrophage inflammatory protein 1a)	[105]
CD154	[106, 107]
IFN-*γ*	[49, 106, 107]
NF-*κ*B	[103]
NKG2D	[108, 109]
PD-L1	[110]
MHC	[103, 111–114]
IL-10	[106, 107, 115–117]
Antigen processing proteins	[113, 118]
IL-1*β*	[49, 115]
IL-6	[115]
IL-12	[49]
TNF-*α*	[49]
